# COMT polymorphism influences decrease of ovarian follicles and emerges as a predictive factor for premature ovarian insufficiency

**DOI:** 10.1186/1757-2215-7-47

**Published:** 2014-05-02

**Authors:** Emerson Barchi Cordts, Monise Castro Santos, Carla Peluso, Erika Azuma Kayaki, Bianca Bianco, Caio Parente Barbosa, Denise Maria Christofolini

**Affiliations:** 1 Center of Human Reproduction and Genetics – Faculdade de Medicina do ABC, Santo André/SP CEP 09060-650, Brazil; 2 Faculdade de Medicina do ABC, Avenida Príncipe de Gales, 821, Santo André/SP CEP: 09060-650, Brasil

**Keywords:** *COMT*, Estrogen metabolism, Infertility, POI, Polymorphism

## Abstract

**Background:**

Estrogens are important factors in the female reproductive functions and are processed by a number of enzymes along their metabolic pathway. The *COMT* gene constitutes a crucial element in estrogen metabolism and is assumed to be involved in the development of Premature Ovarian Insufficiency (POI). This study aimed to determine whether the presence of the *COMT* Val/Met polymorphism (rs4680) is associated to the risk of developing POI.

**Findings:**

In this case–control study, we evaluated 96 infertile women with POI and 120 fertile women as controls, after obtaining a detailed history of the disease and follicle-stimulating hormone measurements, besides karyotype determination and fragile-X premutation syndrome investigation. *COMT* (Val/Met) genotypes were identified by real time PCR (genotyping *TaqMan* assay), and the results were statistically analyzed. A statistically significant difference was found in the distribution of *COMT* genotypes (p = 0.003) and alleles (p = 0.015) between the POI patients and the control group.

**Conclusion:**

We were able to demonstrate a strong association between the *COMT* Val/Met polymorphism and the risk of premature ovarian insufficiency in the Brazilian women evaluated. However, further studies in larger populations are necessary to confirm these findings.

## Background

Premature ovarian failure (POF) is a disorder with a complicated clinical presentation and course that is poorly defined by its name. POF is classically defined as a process in which the gradual decline of ovarian function results in failure of folliculogenesis before the age of 40 years, elevated FSH and low estradiol levels [1-3]. However, this definition does not take into account the longitudinal progression towards the final menstrual cycle. A scientifically more accurate term for the disorder is “primary ovarian insufficiency” (POI), which can be appropriately modified to describe the state of the ovarian function [4]. Indeed, the process of ovarian senescence in this condition may resemble that of natural menopause, which is preceded by several years by elevated FSH levels and menstrual irregularity [4].

The most common etiologies observed for this condition are chromosomal abnormalities, fragile X premutations and autoimmune causes. Once these were ruled out, we can think that a non-obvious genetic pathway could be implicated in the disease. Several genes have been identified as being expressed in the ovary and are postulated to play a role in ovarian physiology and in maintaining normal homeostasis in the ovarian cycle. Alterations in these genes can be associated with the development of POI [5]. A recently demonstrated example of that is that mutations in estrogen receptors can affect regulatory pathways and have been reported to be positively associated with the development of POI [6-8].

It was demonstrated that the gene *COMT* (catechol-O-methyltransferase - Gene ID: 1312) is expressed in granulosa cells [9], where it acts by processing the estrogen metabolites. The increase of these metabolites can promote cellular atresia, with consequent anovulation. The widely studied Val/Met polymorphism (rs4680) that occurs in *COMT* is characterized by the substitution of valine for methionine at codon 158, and this substitution results in a less active enzyme form, which in turn promotes an increase of metabolites in patients with the mutated allele [9].

The main estrogens, estrone (E_1_) and estradiol (E_2_), can suffer oxidative metabolism at different positions, catalyzed by various cytochrome P450 isoforms. The oxidation produces A-ring metabolites such as catecholestrogens (CEs) by 2-and 4-hydroxylation, and D-ring metabolites by 16 α-hydroxylation [10]. The metabolism of CEs and 16 α-OH-estrogens is catalyzed by phase II metabolic enzymes such as COMT [11], which constitutes a crucial element in estrogen metabolism by regulating the inactivation and elimination of carcinogenic metabolites by converting them into non-carcinogenic methoxyestrogens such as 2-methoxyestradiol (2-ME_2_) and 4-methoxyestradiol (4-ME_2_) [12,13]. 2-ME_2_ is suggested to have a potential physiologic role in follicle homeostasis [5].

Thus, our current hypothesis is based on the fact that COMT converts CEs to 2-ME_2_, and that polymorphisms in the *COMT* gene could interfere in the concentration of 2ME_2_, contributing to POI. So, the aim of this study was to determine whether the Val/Met genetic variation in the steroid hormone metabolism gene *COMT* is associated with Premature Ovarian Insufficiency in Brazilian women.

## Material and methods

### Patients

A total of 216 women were selected to participate in the study. Ninety-six of these women, diagnosed with POI, were recruited from the Human Reproduction and Genetics Center of Faculdade de Medicina do ABC (FMABC), Santo André, SP, Brazil. An important fact about the clinical history of these women is that all of them came to the Center with the wish of achieving pregnancy. All patients were diagnosed in FMABC hospitals, based on two confirmed serum FSH level measurements of > 25 IU/L before the age of 40 years. The serum FSH levels were measured at two separate time-points within a period of two months. The control group included 120 healthy women who had gone into physiological menopause around 48 years of age (mean 46.3 ± 1.36 y), had been fertile, with a normal menstrual history, regular menses (every 25–35 days), no personal or family history of premature or early menopause, and no consumption of oral contraceptives or other hormonal medications at the time of recruitment.

All women underwent a complete clinical examination, along with medical and gynecological history including the reproductive health of the patient’s mother, family history, consanguinity, and any other genetic condition in the family, age of menarche and age of menopause. In the patient group, the mean age was 35.7 (±5.14) years, and the mean age at menopause was 31.5 (±6.59) years. All patients had normal karyotypes, determined by analyzing 40 metaphases, and normal alleles for the *FMR1* gene (implicated in the Fragile-X syndrome). The *FMR1* mutation, the main cause of POI, has a high incidence in the world population. The mean FSH serum value was 75.2 (±40.1) mUI/mL. Patients with a known cause of POI, such as karyotype abnormalities, oophorectomy, chemo or radiotherapy were excluded from the study.

### Methods

#### DNA extraction

Peripheral blood was collected from each patient and control in an EDTA-containing tube. Genomic DNA was extracted from peripheral blood lymphocytes, using the salting-out procedures described by Lahiri & Nurnberger [14]. Clinical data and peripheral blood samples were only collected after explaining the objectives of the study and obtaining a signed informed consent form, as approved by the Faculdade de Medicina do ABC ethics committee (process no. 184/2007).

### *COMT* genotyping

Detection of the *COMT* Val/Met (G/Val; A/Met) polymorphism was performed using *TaqMan* PCR. *TaqMan* primers and probes are commercially available and were purchased from Applied Biosystems® (Foster City, CA, EUA) (rs4680, C__25746809_50). The assays were performed with *TaqMan* Universal Master Mix, using 50 ng of DNA per reaction. The PCR conditions were as recommended by the manufacturer: 40 denaturation cycles at 95°C (15 sec), and annealing/extension at 60°C (1 min). The reactions were performed on a Rotor gene 6000 real-time PCR platform (Corbett, Mortlake, New South Wales, Australia).

### Statistical analysis

The chi-square and the logistic binary regression tests were performed to compare the genotype frequencies (rare homozygous, heterozygous and common homozygous), odds ratios (OR), and confidence intervals (CI). The analyses were made using the SPSS 18.0 (SPSS, Inc., Chicago, IL, USA) program. All *p*-values were two-tailed, and 95% confidence intervals (CIs) were calculated. A *p*-value < 0.05 was considered statistically significant.

## Results

We found a differential distribution of genotypes and alleles between the studied groups. The GG (Val/Val), GA (Val/Met) and AA (Met/Met) genotype frequencies of the *COMT* polymorphism in the POI group were 9.4% (9/96), 52.1% (50/960) and 38.5% (37/96), respectively, while in the control group they were 27.5% (33/120), 39.1% (47/120) and 33.3% (40/120) (p = 0.003), respectively. According to the allelic distribution, allele G and A were present, respectively, in 35.4% and 64.5% of the POI group, and in 47.0% and 52.9%, respectively, of the control group, showing a positive statistical correlation between POI and allele A (p = 0.015, OR = 1.62, 95% CI = 1.10-2.39) (Table 1). Both the POI and the control group were in Hardy-Weinberg equilibrium.

**Table 1:** **Genotype and allele frequencies of the *COMT *** **Val/Met polymorphism in POI patients and a control group**

**Population studied**	**n**	***COMT G/A *** **genotypes**				***COMT *** **Alleles**				***Dominant model***		**p**
		**GG (%)**	**GA (%)**	**AA (%)**	**p**	**G (%)**	**A (%)**	**p**	**OR (95% CI)**	**GG**	**GA + AA**	
POF patients*	96	9 (9.4)	50 (52.1)	37 (38.5)	0.003	68 (35.4)	113 (58.8)	0.015	1.62 (1.10 – 2.39)	9	87	0.0008
Control group*	120	33 (27.5)	47 (39.2)	40 (33.3)		113 (47.1)	127 (52.9)			33	87	

*Sample groups are in Hardy Weinberg equilibrium.

## Discussion

The enzyme COMT is ubiquitously found in various mammalian tissues, with high levels in the liver, kidney, endometrium, breast and granulosa cells [15,16]. A common polymorphism, G/A in codon 158 of *COMT*, leads to a substitution of a valine for a methionine in the gene product, which is linked to low COMT activity, with an up to 4-fold decrease in activity in red blood cells and liver [16]. It has been hypothesized that reduced COMT activity may increase the risk of hormone-dependent diseases by enhancing the serum and tissue levels of estradiol, as well as by the accumulation of catecholestrogens and the subsequent oxidative DNA damage [17].

Several association studies in POI candidate genes have been widely used in the search for susceptibility alleles, but only few definitive associations have been established. These inconsistencies in results probably reflect an actual variation in the underlying association between populations studied and the low penetrance of mutations in these multigenic pathways [18]. In the present study, the patient group was homogeneous and especially selected by the presence of infertility, defined according to the minimum propedeutics of the infertile couple. In addition, all patients were investigated as to other causes of POI, such as chromosome aberrations, and positive findings were used as exclusion criteria.

To our best knowledge, this is the first study that correlates *COMT* polymorphism and premature ovarian insufficiency. Here, we found a positive association of allele A of the studied polymorphism with POI, reflecting on the genotype, as we observed a high frequency of heterozygous and mutated homozygous (p = 0.003) and allelic distribution (p = 0.015). The allele acts in a dominant mode. We suppose that it may produce increased damage to ovarian cells, leading to POI.

The estrogen oxidative DNA damage effect has previously been shown to affect both female and male reproduction [19,20]. The risk of oxidative damage and lipid peroxidation was found to be especially high in steroid-synthesizing tissues, because, in addition to oxidative phosphorylation, they use molecular oxygen. Indeed, it has been shown that free radicals inhibit steroidogenesis by interfering with cholesterol transport to the mitochondria and/or the catalytic function of P450 enzymes, which leads to an increase of lipid peroxidation and to the decline of the antioxidant barrier. Considering males, all these changes can alter the testicular cells, including spermatozoa and, therefore, the sperm production, leading to an alteration in male fertility [21]. In females, it can determine cellular apoptosis, corpus luteum regression and follicular atresia [19,22].

Salih et al. [5] proposed that the metabolite 2-ME_2_ has a potential physiological role in follicle homeostasis: under normal conditions, its level is low in early folliculogenesis, rising (probably to the inhibitory range) with the augmented E production in the fully developed dominant follicle. Modulation of COMT activity and, therefore, the concentration of 2-ME_2_ might be part of the ovarian physiological apparatus. Thus, a pathologic alteration of COMT could lead to a major perturbation of folliculogenesis.

In PCOS (Polycystic Ovary Syndrome), catechol O-methyltransferase overexpression and increased levels of 2-ME_2_ in ovarian granulosa cells represent a mechanism leading to abnormalities of steroidogenesis, follicular arrest, and anovulation [5]. We propose that the opposite occurs in POI. Here, we found that most of the POI patients presented an increased incidence of the low-activity *COMT* allele, promoting decreased formation of 2-ME_2._

As suggested by Salih et al. [5], we believe that disturbed levels of 2-ME_2_ could be associated with increased follicular depletion, eventually leading to follicular arrest. This disturbed pathway seems to be an important mechanism associated with Premature Ovarian Insufficiency in Brazilian women. However, further studies in larger sample sets are needed to confirm these findings.

## Competing interests

None of the authors has any conflict of interest to disclose.

## Authors’ contribution

EBC participated in patients’ evaluation and selection and study design; MS, CP and EAK participated in samples preparation, molecular genetics studies and statistical analysis; BB and CPB participated in manuscript design and DMC participated in the conception of the idea, supervision of the research and manuscript design. All authors read and approved the final manuscript.
